# Supplementary material for: Automatic three-dimensional reconstruction of fascicles in peripheral nerves from histological images
Source: PLoS One. 2020 May 14;15(5):e0233028. doi: 10.1371/journal.pone.0233028 (PMC7224505; doi:10.1371/journal.pone.0233028)
Supplement: S1 Data — (PDF) [file pone.0233028.s001.pdf]

SUPPLEMENTARY INFORMATION

SP1.1 TIMING

The breakdown of the combined processing time of all nerve segments for the methods described in the main text was as follows:

- Registration: 4 hours 15 minutes, ~3 minutes per image pair.
- Detection: 44 minutes, ~20 seconds per image.
- Segmentation (H&E): 38 minutes 52 seconds, ~30 seconds per image.
- Segmentation (IHC): 6.5 seconds, < 1second per image.
- Reconstruction: 16 minutes 8 seconds, ~ 8 seconds per image pair.

This timing was obtained using an Intel® Xeon® Processor E3-1240 v2, 3.4GHz, 16GB RAM. Thus, the total processing time for H&E images was approximately 6 hours, the bulk of which was registration. The total processing time for IHC images was approximately 4.5 hours. Note that since each section had a different number of images, the processing time was different for each segment.

SP1.2 ADDITIONAL FIGURES

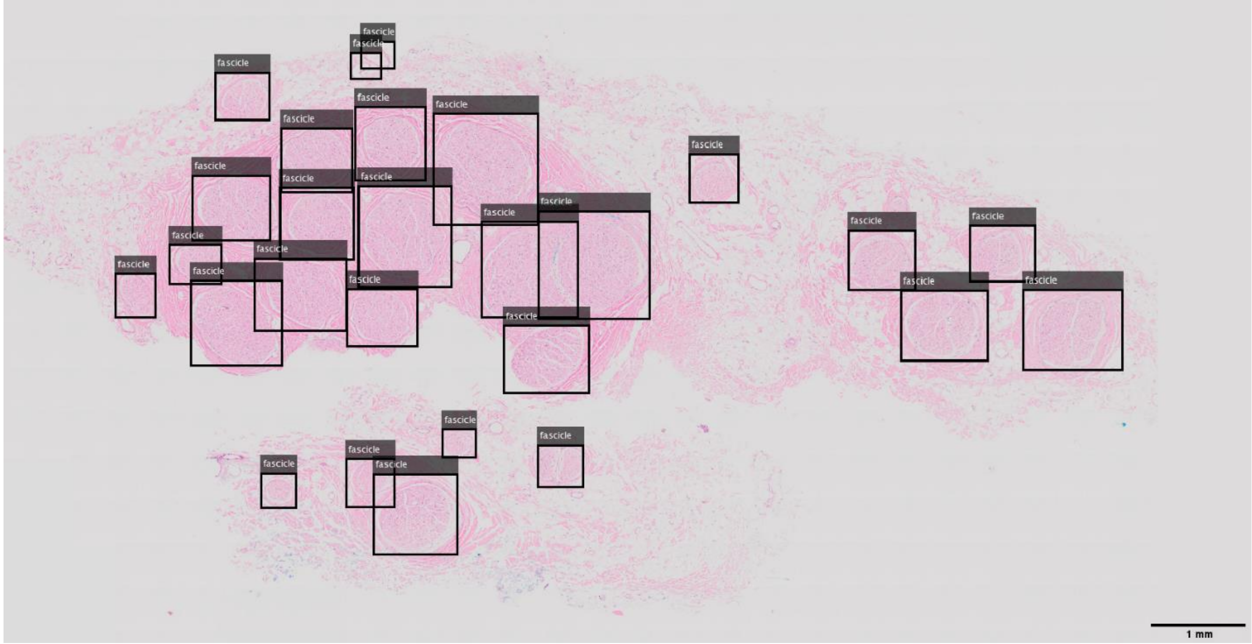

Figure S1: A sample detection. The width and height of each bounding box were used to generate an oval with slightly larger dimensions. The connective tissue outside of the circle was segmented out. Note the two false positive blood vessels detected at the top of the image.

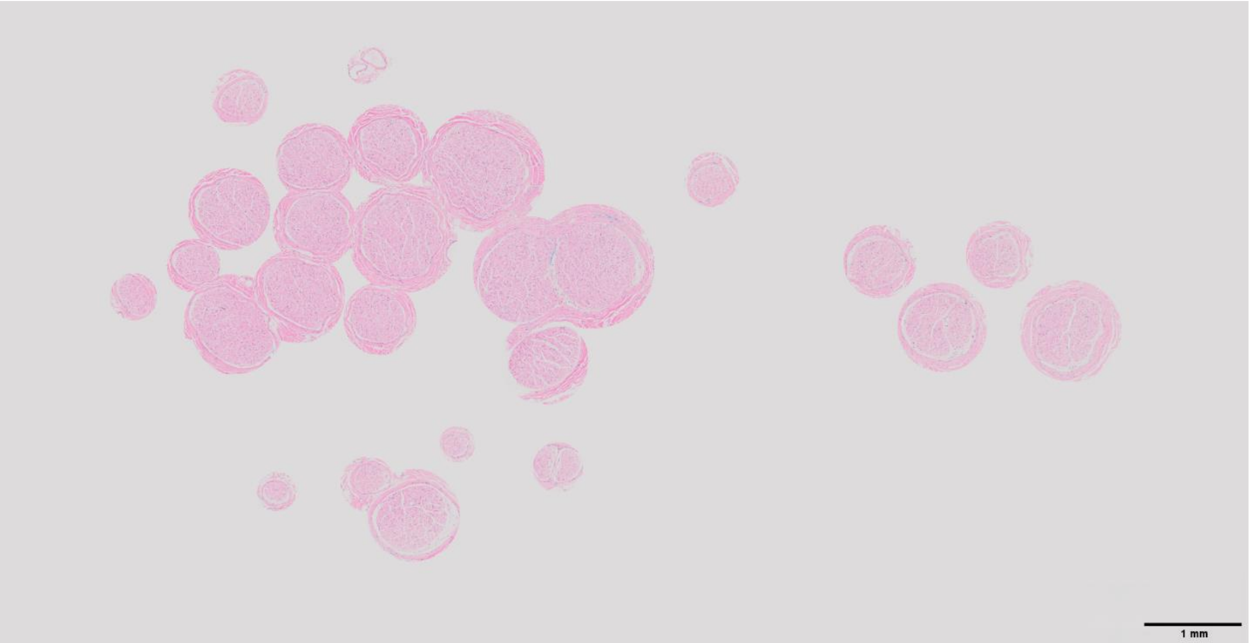

Figure S2: The result after segmenting out connective tissue. Some connective tissue still remains, as the circle was chosen to be conservative so as to not lose any fascicular tissue.

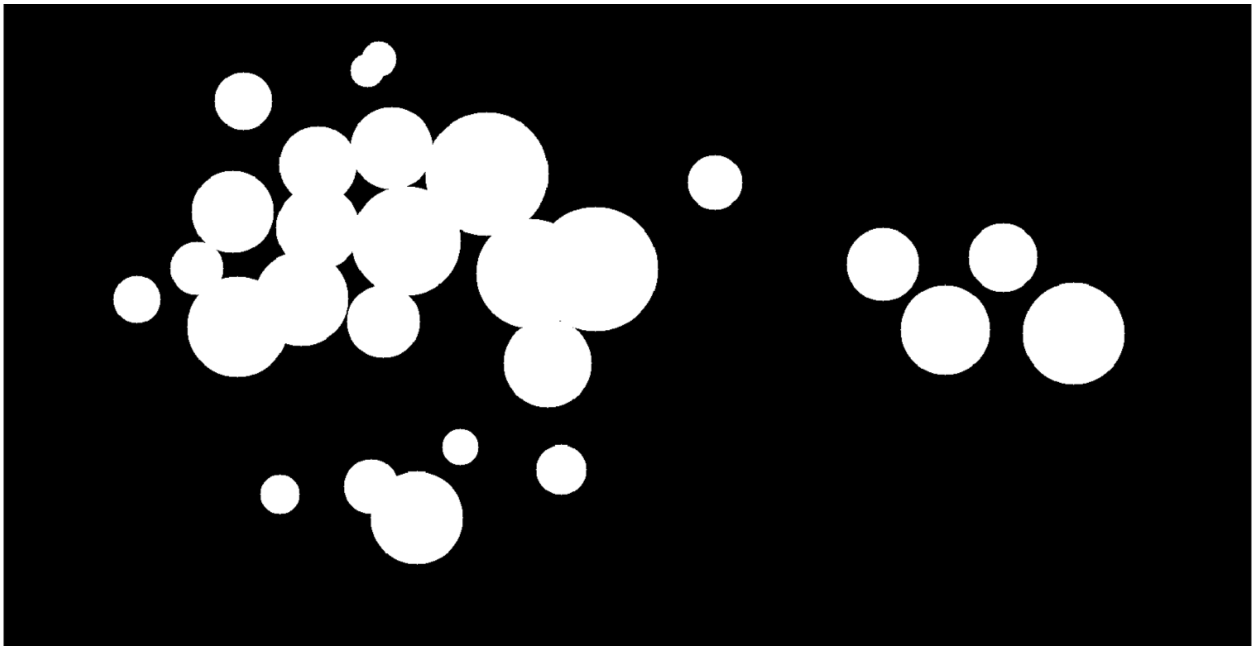

Figure S3: The initial mask used for segmentation.

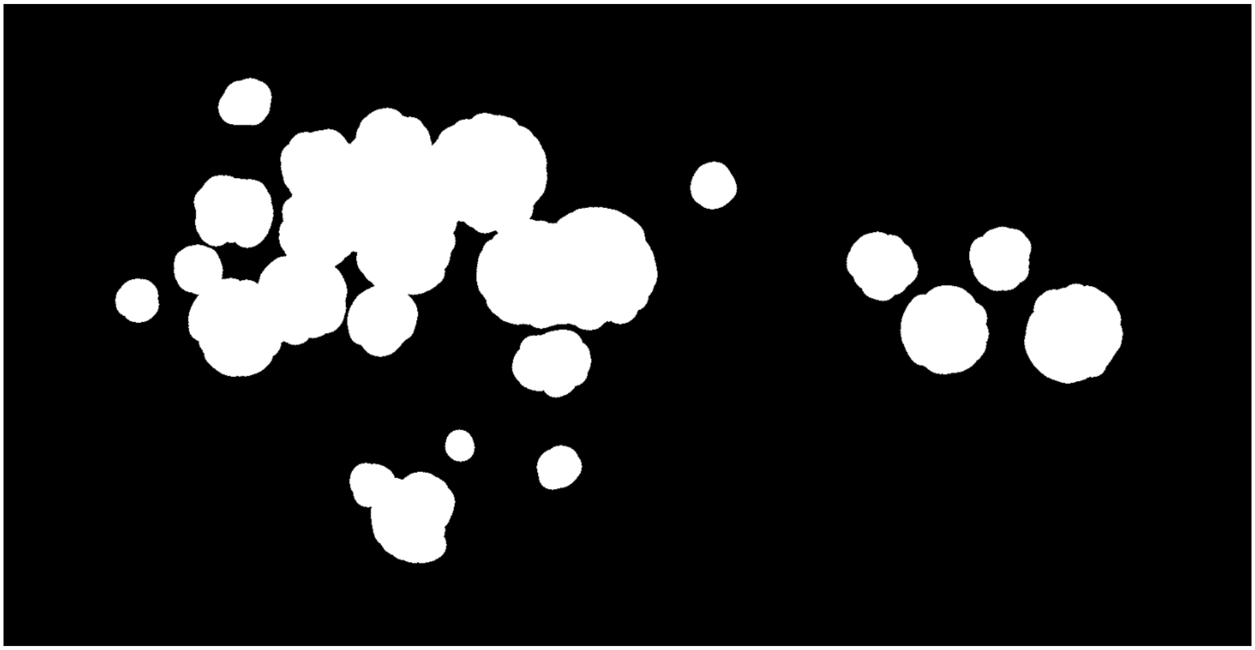

Figure S4: The final mask after segmentation. The boundaries better conform to the shapes of the fascicles.

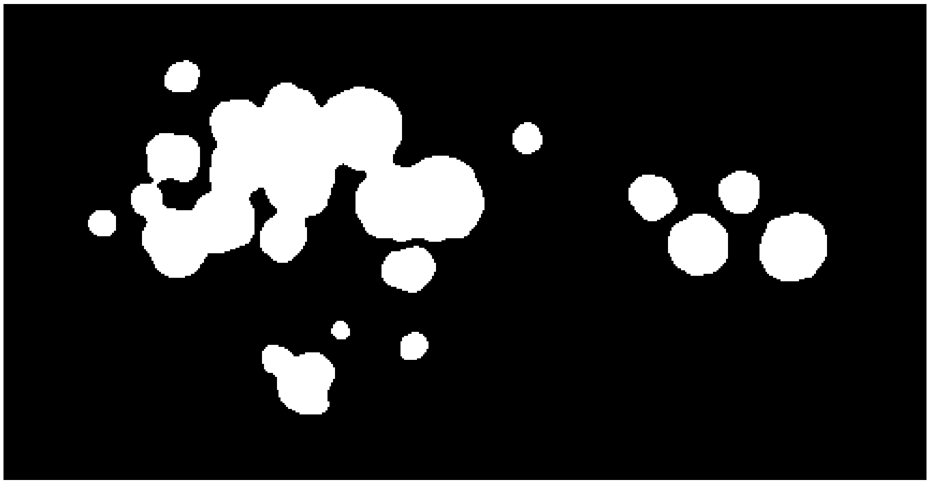

Figure S5: The final mask is scaled down for reconstruction, to save on file size and processing time.

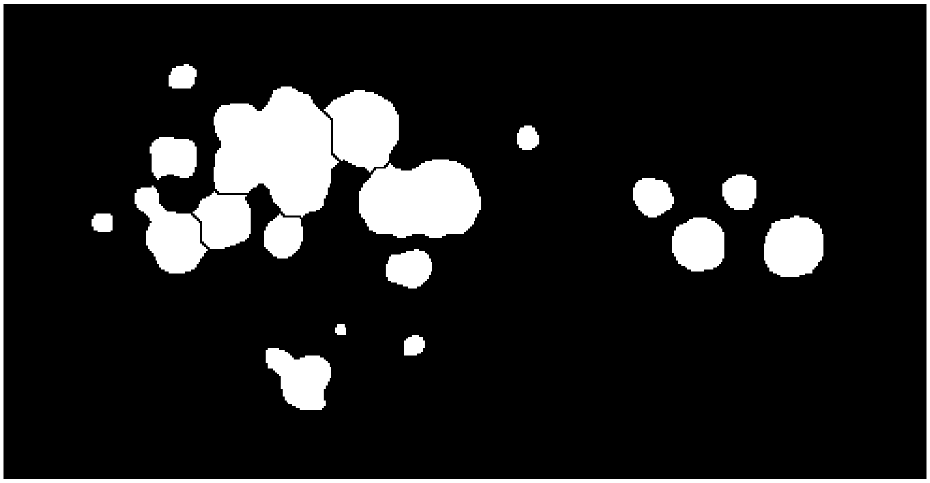

Figure S6: The mask just before reconstruction, after the watershedding, erosion, and a second watershedding. Note that some merged fascicles have been split.

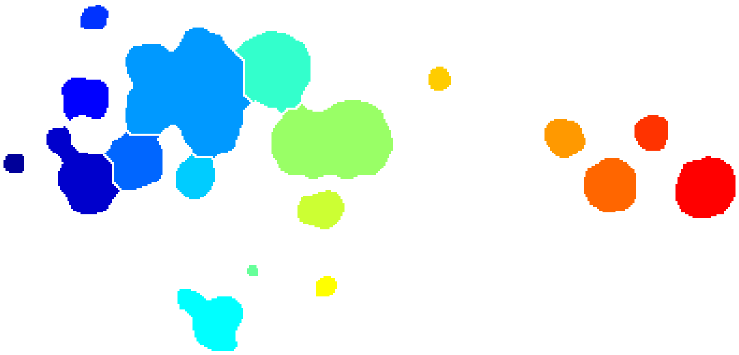

Figure S7: A labelling shows how each fascicle is separated.

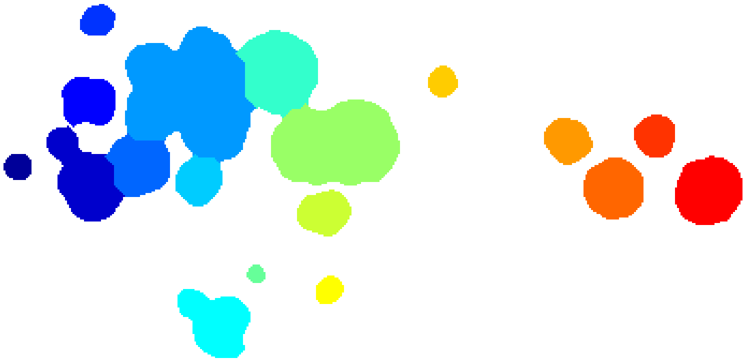

Figure S8: The separate labels persist after the fascicles are dilated back to their original sizes.

SP1.3 DATA REPOSITORIES

The code used in this project can be found at:

https://github.com/dtovbis/FascicleReconstruction/

The image files and trained neural networks can be found at:

https://dataverse.scholarsportal.info/dataverse/FascicleReconstruction
