# Supplementary figures and images for: Automatic three-dimensional reconstruction of fascicles in peripheral nerves from histological images
Source: PLoS One. 2020 May 14;15(5):e0233028. doi: 10.1371/journal.pone.0233028 (PMC7224505; doi:10.1371/journal.pone.0233028)

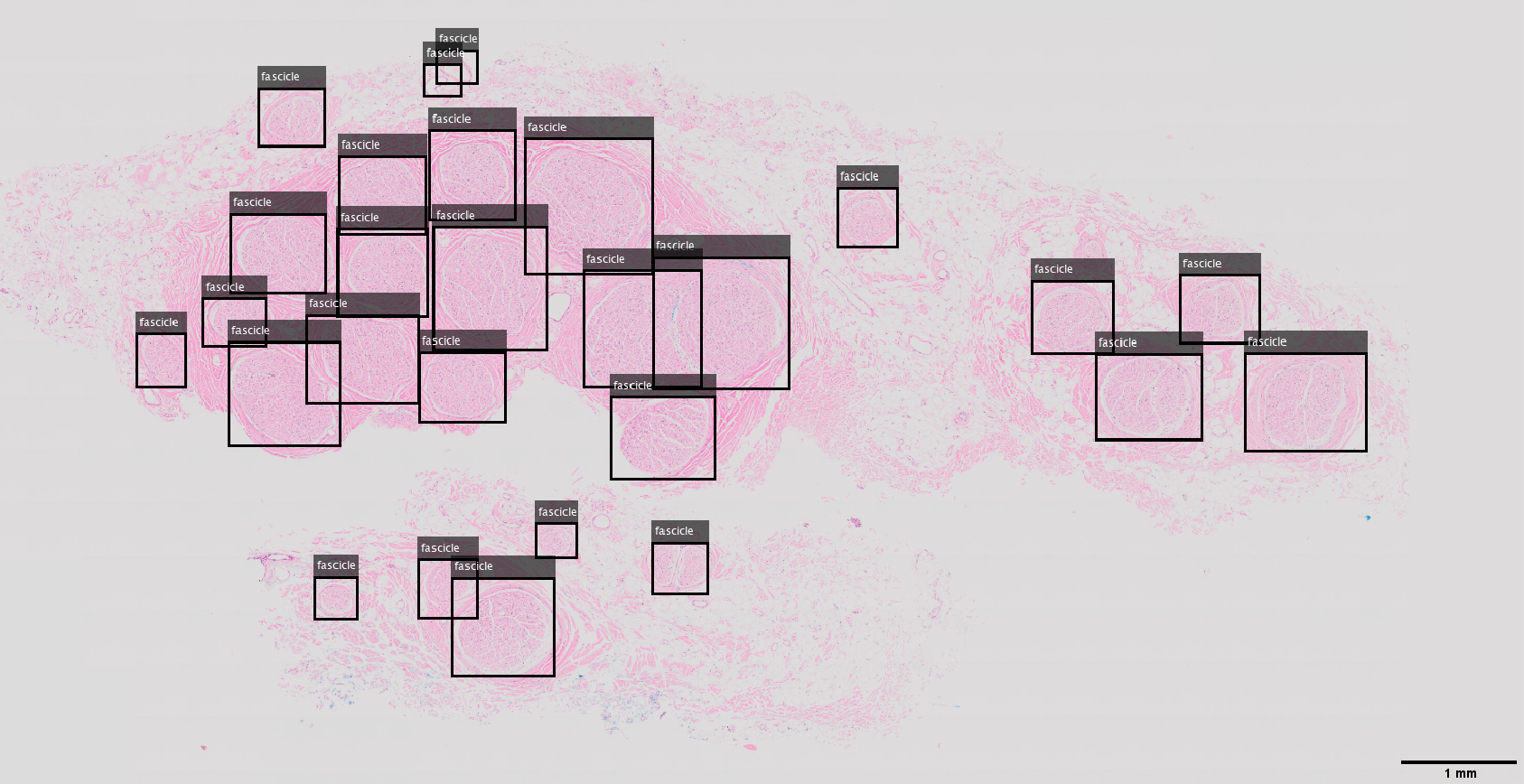

Supplement: S1 Fig — The width and height of each bounding box were used to generate an oval with slightly larger dimensions. The connective tissue outside of the circle was segmented out. Note the two false positive blood vessels detected at the top of the image. (TIF) [file pone.0233028.s002.tif]

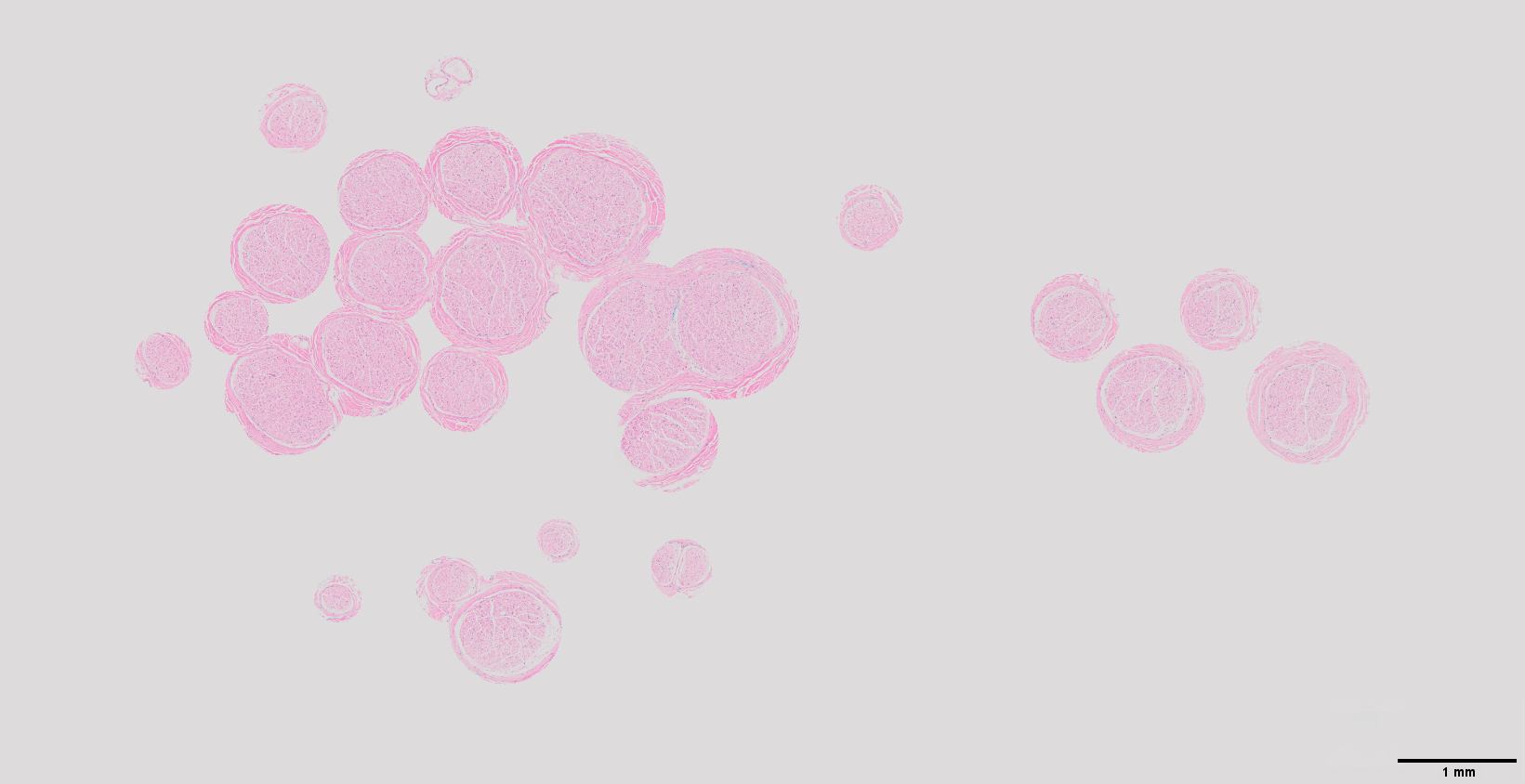

Supplement: S2 Fig — Some connective tissue still remains, as the circle was chosen to be conservative so as to not lose any fascicular tissue. (TIF) [file pone.0233028.s003.tif]

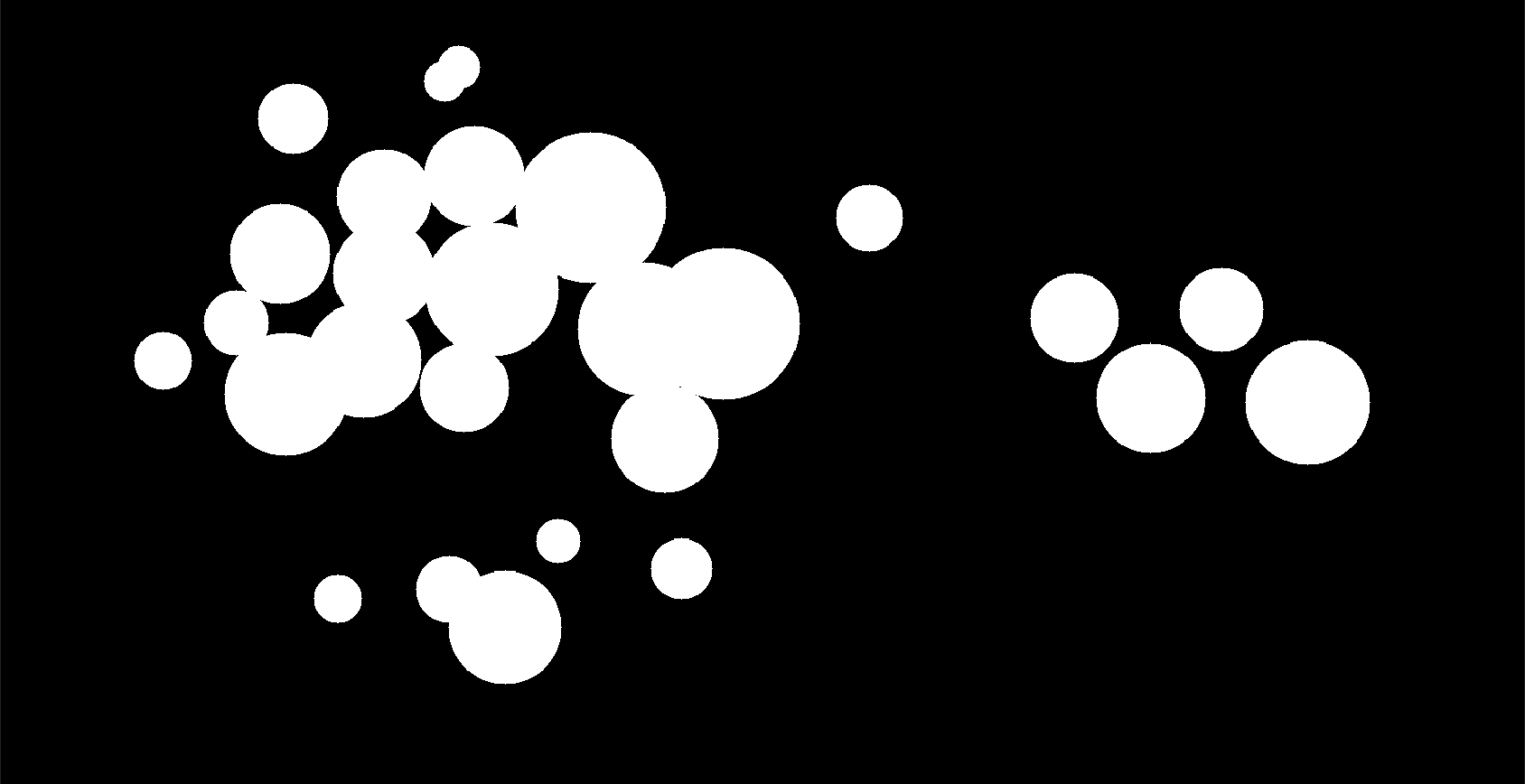

Supplement: S3 Fig — (TIF) [file pone.0233028.s004.tif]

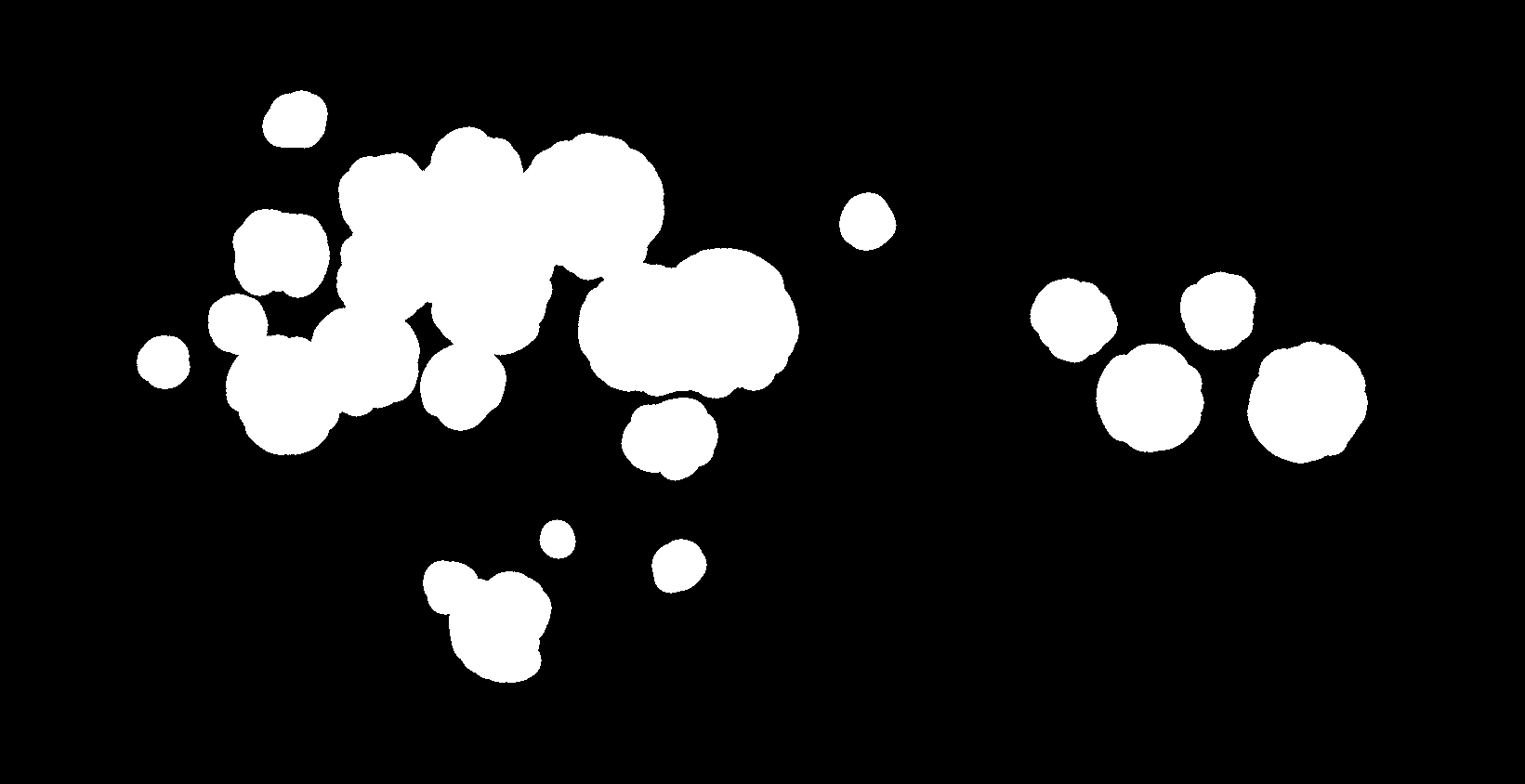

Supplement: S4 Fig — The boundaries better conform to the shapes of the fascicles. (TIF) [file pone.0233028.s005.tif]

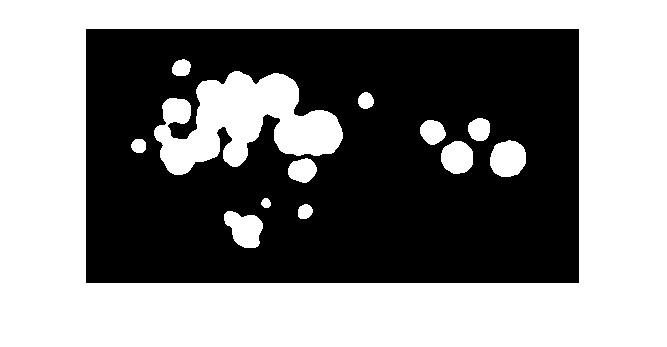

Supplement: S5 Fig — (TIF) [file pone.0233028.s006.tif]

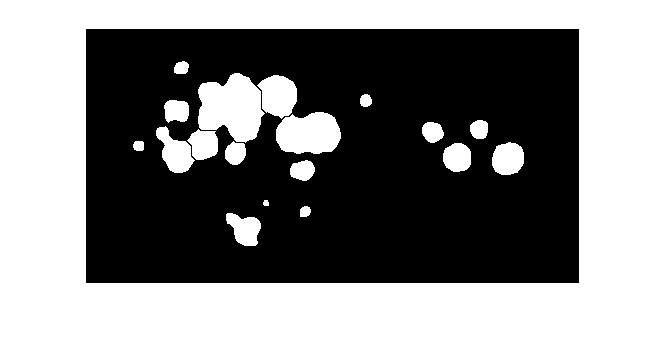

Supplement: S6 Fig — Note that some merged fascicles have been split. (TIF) [file pone.0233028.s007.tif]

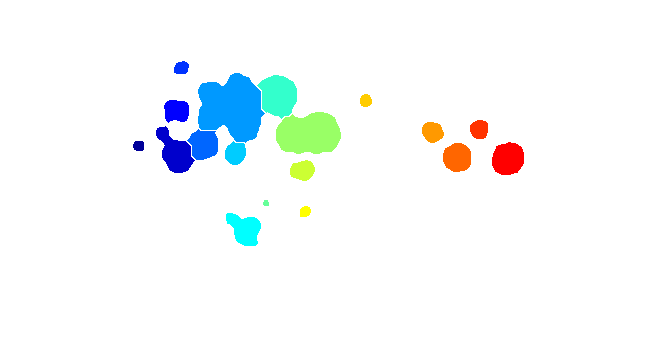

Supplement: S7 Fig — (TIF) [file pone.0233028.s008.tif]

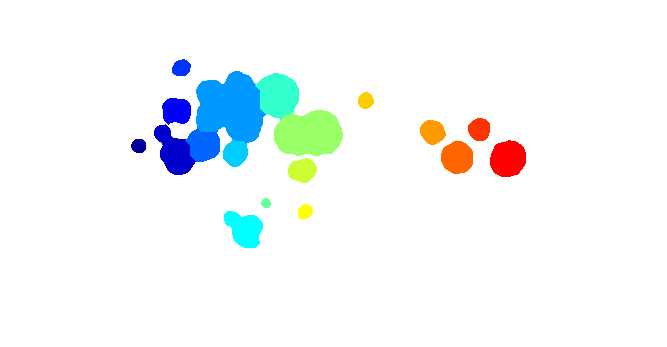

Supplement: S8 Fig — (TIF) [file pone.0233028.s009.tif]
